# Addressing the Experiences of Family Caregivers of Older Adults During the COVID-19 Pandemic in Finland

**DOI:** 10.1177/07334648221095510

**Published:** 2022-05-11

**Authors:** Roosa-Maria Savela, Tarja Välimäki, Irma Nykänen, Sohvi Koponen, Anna Liisa Suominen, Ursula Schwab

**Affiliations:** 1 Department of Nursing Science, University of Eastern Finland, Kuopio, Finland; 2 Institute of Public Health and Clinical Nutrition, University of Eastern Finland, Kuopio, Finland; 3 Institute of Dentistry, School of Medicine, University of Eastern Finland, Kuopio, Finland; 4 Department of Oral and Maxillofacial Diseases, Kuopio University Hospital, Kuopio, Finland; 5 Department of Medicine, Endocrinology and Clinical Nutrition, Kuopio University Hospital, Kuopio, Finland

**Keywords:** caregiving, COVID-19, loneliness, social isolation

## Abstract

This cross-sectional study assessed the experiences of family caregivers of older adults during the COVID-19 pandemic. Participants were recruited (*n* = 101) between April and December 2019. We applied a mixed-method approach. Quantitative data were analyzed using an independent samples t-test and logistic regression analysis, and qualitative experiences with modified thematic content analysis. The mean age of the family caregivers was 76 years (*SD* = 7), and 72% were females. Experiences of loneliness and worry during the pandemic were evaluated by self-assessment. Approximately one-third of the participants reported loneliness and worry. These experiences were further associated with female sex, increased psychological distress and depressive symptoms, and decreased physical condition and social relationships. Family caregivers were also worried about the pandemic’s impact on health and well-being. Thus, the COVID-19 pandemic has added an extra psychosocial load to family caregivers. The post-pandemic era requires increased attention to re-evaluating policies and services.

***What this paper adds***

- Our study adds to the existing literature by assessing how social isolation due to the pandemic has affected the well-being of family caregivers who take care of older adults.
- The COVID-19 pandemic and its measures and regulations have created new challenges and needs for family caregivers.
- The pandemic has added extra mental and psychosocial loads to older family caregivers, including worry and a sense of loneliness.

***Applications of study findings***

- There is a need to enhance and re-evaluate the policies and support services regarding socially vulnerable populations.
- Implementing community-based programs, ensuring support services, providing mental health facilities through online services, and implementing a stronger life-course approach in healthcare to maintain family caregivers’ well-being should be considered.

The coronavirus (COVID-19) pandemic has changed our lives and has caused several adverse effects on health and well-being. It is well documented that the impacts have been significant on individuals’ social relationships, and physical and mental health (Beach et al., 2021; Zaninotto et al., 2022). The consequences of the pandemic have been noteworthy in vulnerable populations, increasing existing health inequalities (Dorn et al., 2020).

COVID-19 can be severe for persons of any age. However, older adults are at a greater risk of serious illness and death (Verity et al., 2020). Therefore, the pandemic has forced measures and regulations to limit the spread of the virus to protect vulnerable populations. These regulations have meant the physical distancing and social isolation of older adults (Armitage & Nellums, 2020; Tuijt et al., 2021). However, while physical distancing is effective in infection prevention (World Health Organization, 2020), it has caused mental health outcomes. In short, social isolation among older adults has been defined as a severe public health concern (Armitage & Nellums, 2020). Hence, their social isolation may lead to poorer mental health and well-being, which is in turn linked to a decline in physical health and cognition (National Academies of Sciences, Engineering, and Medicine, 2020). Therefore, the situation has increased interest in the well-being of older family caregivers (FCs).

Partners, relatives, friends, or neighbors to individuals with physical, mental, or cognitive challenges usually provide family caregiving (Schulz et al., 2020). Unfortunately, several studies have shown that some caregivers have poorer health outcomes than non-caregivers. These outcomes include lower quality of life (Välimäki et al., 2016), higher rates of psychological distress (Schulz et al., 2020), and poorer physical and mental health (National Academies of Sciences, Engineering, and Medicine, 2016).

The pandemic and the resulting social isolation may have worsened FCs’ well-being. Thus, prior evidence shows that loneliness and social isolation are associated with negative health outcomes (National Academies of Sciences, Engineering, and Medicine, 2020). However, social isolation is distinct from loneliness. In brief, social isolation refers to “the objective situation of being alone or lacking social relationships” (Perlman & Peplau, 1998, p. 571). In contrast, loneliness is commonly defined as “the subjective psychological discomfort experienced by people when their network of social relationships is significantly deficient in either quality or quantity” (Perlman & Peplau, 1998, p. 571). Sometimes, loneliness is divided into two key dimensions: emotional and social loneliness (Perlman & Peplau, 1982; Weiss, 1973), where emotional loneliness refers to a lack of close emotional attachment, and social loneliness refers to the absence of an adequate social network (Weiss, 1973).

Previous evidence shows that loneliness in older adults is complex. Several factors, including increasing age, income, health status, place of residence, and contact with friends and family, affect loneliness (Drennan et al., 2008). Other identified risk factors include experiences of depression, not being married/partnered, and partner loss (Dahlberg et al., 2022). In addition, some researchers have examined the association between loneliness, social isolation, and family caregiving. For example, spousal caregivers might experience more loneliness, depression, and lower life satisfaction than non-caregivers (Wagner & Brandt, 2018). In addition, caregivers of those with dementia may have higher odds of depressive symptoms compared to non-caregiving partners, partly mediated by loneliness (Saadi et al., 2021). In addition, a prior study indicated that caregivers of those with dementia have greater social isolation and increased caregiving stress associated with loneliness (Victor et al., 2021).

However, the loneliness and distress of FCs of older adults have not been explicitly evaluated in previous intervention reviews (Gorenko et al., 2021). Moreover, there is still limited evidence of FCs’ concerns, loneliness, and social support during COVID-19. Some pandemic-related evidence has focused on FCs caring for a person affected by Alzheimer’s disease and other forms of dementia (Frangiosa et al., 2020) and assessed differences between subgroups of caregivers and non-caregivers (Park, 2021). Similarly, pandemic-related evidence has assessed caregivers’ self-efficacy and stress (Sheth et al., 2021) and used a quantitative research method to assess the pandemic’s effects (Beach et al., 2021).

Our study adds to the existing literature by assessing how social isolation due to the pandemic has affected the well-being of FCs who take care of older adults with various health conditions. The primary aim was to assess FCs’ experiences during the pandemic. We used a mixed-methods study design and examined FCs’ experiences regarding loneliness, worry, social support, and related factors. These factors include sociodemographic features (e.g., rural and urban municipalities), psychological distress, depressive symptoms, and quality of life.

## Methods

This cross-sectional study used a mixed-method approach to assess FCs’ experiences during the COVID-19 pandemic. This study is part of the randomized population-based multidisciplinary lifestyle, nutrition, and oral health in caregivers (LENTO) study (Nykänen et al., 2021). We did not initially propose to assess the effects of COVID-19. However, questions on the experiences of loneliness, worry, and social support were included when the first wave of the pandemic and social isolation started in Finland (March 2020). Thus, there was an impression of dramatic changes in the lives of older adults.

### Sample

We recruited participants between April and December 2019 from two municipalities in the northern Savo Province, Finland. The inclusion criteria of FCs were those who lived in the municipality of Kuopio (urban municipality) or Vesanto (rural municipality), had a valid care allowance granted by municipalities, and took care of a person aged 65 or older. However, FCs who took care of care recipients (CR) with end-of-life care were excluded. Otherwise, there were no exclusion criteria regarding the study participants’ maximum age, morbidity, or cognition.

**Figure 1.**
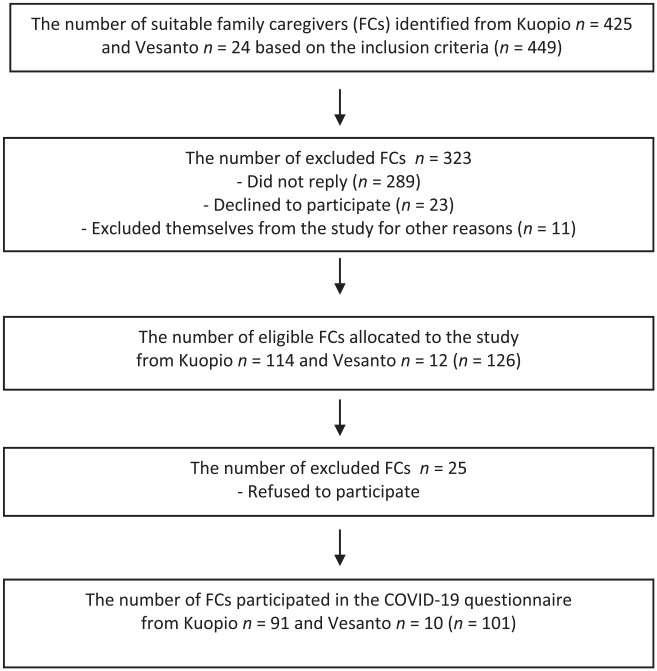
The STROBE (Strengthening the Reporting of Observational Studies in Epidemiology) flow chart of the study sample.

We recruited participants in collaboration with the municipalities’ social and healthcare workers. Thus, all FCs with a care allowance are in the municipalities’ registers. The municipality workers provided addresses of the eligible population based on the inclusion criteria. The research team sent letters to these FCs. Of the 449 eligible participants, 126 agreed to participate in the study. Of this population, 101 (80%) answered the COVID-19 questionnaire (Figure 1).

### Data Collection

This study collected the quantitative and qualitative data in Finnish. These included questions on sociodemographic factors and validated questions on psychological distress, depressive symptoms, and quality of life. In addition, researchers provided questions on experiences regarding loneliness, worry, and social support. These questions were not pilot tested. The duration of the interviews was approximately 30 minutes. A trained study nurse and other trained members of the research team performed these measurements.

We collected both quantitative and qualitative data. Therefore, the checklists of Strengthening the Reporting of Observational Studies in Epidemiology (STROBE) and Consolidated Criteria for Reporting Qualitative Research (COREQ) were followed (Tong et al., 2007; Vandenbroucke et al., 2007). Data collection began during the first and second pandemic waves, between June and December 2020, at FCs’ households. We used personal protective equipment and maintained social distancing during home visits. However, some interviews were also conducted outdoors because of the participants’ wishes.

### Measurements

#### Sociodemographic Factors

The study nurse collected information on the FCs’ background factors, including age, sex (female/male), and area of residence (Kuopio/Vesanto).

#### Experiences during the COVID-19

We interviewed FCs based on their experiences regarding the COVID-19 pandemic. We had four dichotomous questions (yes/no), and some of them were followed by an open-ended question. The four questions were (1) “Have you felt loneliness during the COVID-19 restrictions?” (*n* = 101), (2) “Has your loneliness increased during the COVID-19 restrictions?” (*n* = 72, response rate 71%), (3) “Are you worried about the situation regarding the COVID-19 pandemic? (*n* = 101) If yes, what specifically?” and (4) “Have you received (social) support during the COVID-19 restrictions? (*n* = 101) If not, what kind of support would you have needed?” The authors used these questions to obtain a diverse perspective on FCs’ experiences.

#### Psychological Distress

The psychological distress of FCs was assessed with a valid and reliable General Health Questionnaire (GHQ-12), which includes 12 statements on a four-point scale from 0 (not at all) to 3 (more than usual) (Goldberg & Williams, 1988). The maximum score was 36. Higher scores indicated psychological distress.

#### Depressive Symptoms

We assessed depressive symptoms of FCs using the 15-item Geriatric Depression Scale (GDS-15) (Yesavage & Sheikh, 1986) with higher scores indicating mild to severe depression. Therefore, we considered scores from 0 to 4 as normal.

#### Quality of Life

FCs were interviewed based on their quality of life (QoL) using the World Health Organization Quality of Life (WHOQOL)-BREF questionnaire, a shortened version of the WHOQOL-100 questionnaire (World Health Organization, 1998). The WHOQOL-BREF includes 26 questions and covers all four domains of QoL: (1) physical health, (2) psychological health, (3) social relationships, and (4) environment.

### Data Analysis

#### Quantitative analysis

First, we performed descriptive analyses to summarize the results using numbers, percentages, means (M), and standard deviations (SD). Before data analyses, we examined the normality of data variables using the Kolmogorov-Smirnov test. Then, statistical comparisons between the characteristics were made using the independent samples *t*-test or alternative test (i.e., Mann-Whitney U test). In addition, binary logistic regression, expressed in odds ratios, was performed to identify characteristics and the association of QoL domains with the four COVID-19 questions. The dichotomous answers from the COVID-19 questions were the outcomes, and QoL domains and psychological distress were predictors in the binary logistic regression. We adjusted for age and sex. A *p-*value of .05 or less was significant, with a 95% confidence interval (CI). We identified only a few missing values, without specific patterns. The data analysis was completed using SPSS statistical software (IBM SPSS Statistics for Windows, version 26.0).

### Qualitative analysis

Open-ended answers were analyzed using modified thematic content analysis (Braun & Clarke, 2006; Vaismoradi et al., 2013). This approach was used to identify, analyze, and report identified patterns (themes) in the data (Braun & Clarke, 2006). In the first phase of the analysis, open-ended answers were listed and read to understand the content. Then, the data were organized to see patterns in the content; the 21 identified codes were used to compare similarities and differences regarding the answers. Next, one researcher (T.V) formulated the themes based on the initial coding and the relation of the codes. The same researcher coded the interviews and conducted the analyses. Finally, the analysis formulated descriptive themes which were translated into English for reporting purposes.

## Results

### Description of the Sample

A total of 101 FCs participated in this study. Their mean age was 76 years (*SD* = 7), and male FCs were significantly older (*p = .*041). Most of the FCs were female (72%) and lived in Kuopio (90%). Based on the descriptive analysis, approximately 27% of FCs experienced loneliness during the pandemic. However, a sense of loneliness occurred more frequently among female than male FCs (*p* = .024). Moreover, 34% of FCs worried about the pandemic, and 36% experienced an increased sense of loneliness. Nevertheless, there were no municipality-based differences in the sense of loneliness or worry. Please see Table 1.

**Table 1.** Characteristics of family caregivers.

Sociodemographic Characteristics		*n =* 101
Females, *n* (%)		73 (72%)
Area of living, *n* (%)	Kuopio	91 (90%)
	Vesanto	10 (10%)
Age, mean (SD), years		76 (7)
	Females	75 (6)
	Males	78 (7)
*Experiences regarding the COVID-19 pandemic, n (%)*		*n =* 101
Have you felt loneliness during the COVID-19 restrictions?		
	Yes	27 (27%)
	No	74 (73%)
Has your loneliness increased during the COVID-19 restrictions? (*n=*72)		
	Yes	26 (36%)
	No	46 (64%)
Are you worried about the situation regarding the COVID-19 pandemic?		
	Yes	34 (34%)
	No	67 (66%)
Have you received (social) support during the COVID-19 restrictions?		
	Yes	52 (51%)
	No	49 (49%)

*Note.* SD = standard deviation. Descriptive statistics for continuous (mean, SD) and categorical variables (*n*, %).

### Quantitative results

The analyses showed that FCs’ sense of loneliness and worry were associated with higher scores for depression and psychological distress, and lower scores for physical health and social relationships in the WHOQOL-BREF. For example, higher scores for depression were associated with a sense of loneliness (*p = .*029), increased sense of loneliness (*p = .*022), and worry (*p* = .006) during the COVID-19 restrictions. The binary logistic regression analysis showed that higher scores for psychological distress (GHQ-12) predicted sense of loneliness (*OR* = 1.17, 95% [CI 1.00, 1.37]) and worry (*OR* = 1.45, 95% CI [1.14, 1.85]) (data not shown). In addition, FCs’ sense of loneliness and worry were associated with a decline in physical health and social relationships in the WHOQOL-BREF. Hence, lower scores for the social domain predicted a sense of loneliness (*OR* = 0.76, 95% CI [0.61, 0.94]) and increased sense of loneliness during the COVID-19 pandemic (*OR* = 0.76, 95% CI [0.60, 0.96]). In addition, lower scores for the physical domain (*OR* = 0.81, 95% CI [0.66, 0.99]) predicted the FCs’ worry about the situation. The environment or psychological health domains did not predict a sense of loneliness, worry, or social support (Table 2).

**Table 2.** Associations between family caregivers’ four domains of quality of life and the COVID-19 questions.

Questions	QoL Domains	B	Sig.	OR	95% CI for OR
Sense of loneliness during the COVID-19^1^	Physical	0.038	0.739	1.038	0.832 to 1.296
	Psychological	0.110	0.493	1.116	0.815 to 1.528
	Social	−0.276	0.012*	0.759	0.612 to 0.941
	Environmental	−0.245	0.149	0.783	0.562 to 1.091
Increased sense of loneliness during the COVID-19^2^	Physical	−0.084	0.509	0.920	0.717 to 1.180
	Psychological	0.053	0.771	1.055	0.737 to 1.511
	Social	−0.277	0.020*	0.758	0.600 to 0.958
	Environmental	−0.083	0.658	0.920	0.637 to 1.330
Worry about the situation during the COVID-19^1^	Physical	−0.212	0.050 *	0.809	0.655 to 0.998
	Psychological	0.231	0.124	1.259	0.939 to 1.689
	Social	−0.001	0.993	0.999	0.825 to 1.211
	Environmental	−0.266	0.100	0.766	0.558 to 1.052
Receiving (social) support during the COVID-19^1^	Physical	−0.056	0.578	0.945	0.775 to 1.153
	Psychological	−0.277	0.056	0.758	0.571 to 1.007
	Social	0.084	0.354	1.088	0.911 to 1.299
	Environmental	0.184	0.214	1.202	0.899 to 1.608

*Note.* Analyses were conducted using binary logistic regression analysis.

CI = Confidence Interval. OR = Odds Ratio. QoL= Quality of Life.

1 *n* = 101.

2 n = 72.

* *p*-value ≤ .05.

### Qualitative results

#### Experiences of worry

The qualitative results were based on two open-ended questions. The first open-ended question was, ”Are you worried about the situation regarding the COVID-19 pandemic? If yes, what specifically?” FCs’ answers were linked to three identified themes (Table 3).

**Table 3.** Themes and subthemes expressing family caregivers’ worries during the social isolation.

Themes	Subthemes
1. Impact of unpredictable epidemic	Dangers of the COVID-19
	Uncertainty
2. Belonging to a risk group	Fear of the COVID-19 infection
	The anxiety of care recipient’s illness
	The impacts of restrictions
3. Emotional and physical isolation	Being alone at home
	Constant worry
	Others’ indifferent to the risks of the COVID-19

The first theme included FCs’ awareness of COVID-19 and anxiety about its severity. FCs are knowledgeable of threats that infection causes, which further increases uncertainty in everyday life. They expressed concern about how society and the healthcare sector would manage through the unpredictable pandemic. They also reported that their CR’s functional ability worsened during the stay-at-home restrictions. Examples of statements made by participants regarding their worry included:

“[Worry about] Spread of the COVID-19 infection and its unpredictability, and the pandemic.”

“[Worry about] How will the COVID-19 spread? Will there be another wave?”

“[Worry about] A disease that is unprecedented.”

The second theme included FCs’ understanding of being at risk for COVID-19. They were concerned about getting the infection themselves or the CR. Hence, in FCs’ minds, the potential risks and fatality of the infection were evident. They feared getting a potentially fatal illness themselves. Several expected concerns also arose among FCs about contracting the disease; they were fearful of infecting loved ones. These thoughts led to common fears about the CRs’ future. Some FCs have reported that their CR depends on their health and well-being. In addition, FCs occasionally placed CRs in short-term care during the pandemic. FCs were anxious that CR could contract COVID-19 during care. Examples of statements made by participants regarding the potential of getting or spreading COVID-19 to CRs included:

“I am part of the risk group. My husband is also part of the risk group, and I am afraid for him.”

“What if I fall ill and infect my husband?”

“What if my spouse contracts the virus and might not survive it?”

The third theme focused on the emotional and physical isolation of FCs. Constantly staying at home made FCs feel lonely. Coping day after day was exhausting and was associated with the fear of getting depressed. FCs were still longing to meet relatives and grandchildren, but the constant worry was present. Other people’s indifference to guidelines and recommendations to stay at home also irritated some FCs. They responded by naming loneliness and coping when asked what especially worried them.

### Experiences of support

The second open-ended question was: “Have you received (social) support during the COVID-19 restrictions? If not, what kind of support would you have needed?” Most FCs did not require any specific extra support. However, some of the FCs would have needed more companionship, days off from caregiving, and food delivery at home.

### Study Integration

Quantitative and qualitative data provided related evidence. For example, both analyses showed similar themes, including depressive symptoms, fear of depression, psychological distress, coping, a decline in physical health and social relationships, and emotional and physical isolation. However, the qualitative data provided a more detailed description. For example, the descriptive analysis revealed that approximately one-third of the FCs were worried about COVID-19. The qualitative analysis showed that they were worried about loneliness, coping abilities, depression, COVID-19 infection, and unpredictable consequences of the pandemic. Similarly, quantitative data showed that around 50% of FCs did not receive any support during the pandemic. Again, qualitative data showed that FCs would have needed some services, including days off from caregiving, food delivery at home, and companionship.

## Discussion

Approximately one-third of FCs reported loneliness and worry during social isolation, which were associated with female sex, increased psychological distress and depressive symptoms, and decreased physical health and social relationships. Moreover, FCs were aware of the consequences of COVID-19. Thus, FCs understood that they were at risk and were anxious about its severity. They further experienced emotional and physical isolation.

Our evidence shows that the COVID-19 pandemic and its measures and regulations have created new challenges. The pandemic has added extra mental and psychosocial loads to older FCs. Similar results were found in a longitudinal cohort study of older adults in England (Zaninotto et al., 2022). Their well-being and mental health were affected by COVID-19. However, social isolation may have influenced those already in a vulnerable position.

In addition, our evidence showed that FCs required more companionship and days off from caregiving. This is in line with previous evidence showing that the pandemic has increased FCs’ burden compared to the pre-pandemic period (Archer et al., 2021). Our findings suggest that the Finnish government and municipalities have provided limited support for FCs to maintain caregiving during the pandemic. Many services were temporarily discontinued or deteriorated in Finland. This suspension of services may show a lack of preparedness by the healthcare system to support vulnerable populations in exceptional circumstances. In addition, there may have been limited healthcare guidelines. For example, Finnish municipalities were less likely to provide new online technologies to support older adults during social isolation (Eronen et al., 2020). In addition, some online services tailored for FCs were unsuitable and failed to deliver support during the pandemic (Eronen et al., 2020).

### Recommendations

#### Policy Strengthening

While the Finnish Support for Informal Care Act of 2016 states that “The municipality must, if necessary, arrange welfare and health examinations for the caregiver and social and health services that support his or her well-being and care duties” (Act on Support for Informal Care, 2005), FCs’ rights and support may not be fully covered. Hence, the current Act may lead to the unequal provision of FC services by different municipalities. Furthermore, the statute does not require municipalities to provide ongoing support or mental health services without FC’s demand or identified needs. However, previous evidence shows that older adults struggle to seek mental health services because of several barriers, namely stigma, poor mental health literacy, and identification of mental health challenges as a natural part of aging (Pywell et al., 2020; Titov et al., 2016). Therefore, as part of the post-pandemic acts, it is necessary to strengthen the statutes regarding socially vulnerable populations. For instance, in Finland, it is necessary to consider removing ambiguity (i.e., *“if necessary”*) in the Act. Hence, strengthening laws related to healthcare services could reduce barriers to seeking help for mental health and improve social inclusion and health equity.

#### Community-based Programs

Our evidence shows that FCs experienced emotional and physical isolation and a decline in social relationships. Therefore, FCs should be more strongly integrated into society, communities, and support services as a post-pandemic act. Thus, previous evidence shows that social integration has a protective impact on morbidity and mortality (Gerst-Emerson & Jayawardhana, 2015). Therefore, community-based and intergenerational programs could ensure greater inclusion in society after the pandemic.

#### A Life-Course Approach

Our evidence showed that some FCs experienced depressive symptoms, fear of depression and coping abilities, and a decline in physical health. Therefore, we need more information regarding the abilities, physical and mental health, and background factors of FCs. Thus, it is necessary to identify their vulnerabilities. This approach requires “a life-course perspective” in healthcare, explicitly recognizing the causal links between exposures and outcomes within an individual’s life course (Solar & Irwin, 2010). In addition, assessing the social determinants of mental health should also be considered (Savela et al., 2022).

#### Online Services

The healthcare sector should consider online services in the post-pandemic era. For instance, FCs of older adults could receive health education, health services, and social support through web-based applications. Hence, technology might increase the effectiveness of caregiving (Schulz et al., 2020). In addition, the technology could provide physical and mental activities for FCs and CRs to maintain their well-being.

### Strengths and Limitations

The strengths of this study include validated measurement tools for FCs’ psychological distress, depressive symptoms, and quality of life. In addition, a trained study team collected data during the social isolation of FCs, and the COVID-19 restrictions were ongoing. This data collection period reduces recall bias in FCs’ experiences regarding their loneliness and worry. The mixed-method research approach also combines both quantitative and qualitative data, balancing the limitations of each method. In addition, the open-ended questions had significant value because the study participants could provide more information on their experiences. However, this study also had several limitations.

First, this was a cross-sectional study, presenting the study participants at one point. This means we cannot draw causal conclusions between FCs’ loneliness and worry and the associated factors. In addition, we did not use validated tools to assess loneliness and worry. Instead, we assessed these experiences using a dichotomous answer format, limiting the assessment of various dimensions and experiences of loneliness and worry. We identified this procedure as a significant limitation of this study. In addition, one researcher translated only the themes of qualitative analysis into English. Moreover, we may have recruited only those FCs who were healthy enough to take part. In addition, CRs were often present during the interviews, which could have affected the FCs’ answers.

Second, we recognize the challenge of interviewing older adults at their homes during the pandemic. Therefore, we ensured the subjects’ agreement to continue home visits. In addition, the study participants were aware of the study process and its purposes. They were also familiar with the home visits by the research team. The research team also had the university’s consent to continue the study.

## Conclusion

FCs have had several negative experiences during the pandemic. We highlight the need to enhance and re-evaluate the policies and support services, including implementing community-based programs, ensuring support services, providing mental health facilities through online services, and implementing a more robust life-course approach in healthcare to maintain FCs’ well-being.
